# Evidence of Avian Predation on a Critically Endangered Elasmobranch, the Halavi Guitarfish (*Glaucostegus halavi*), in the Red Sea

**DOI:** 10.1002/ece3.73120

**Published:** 2026-03-09

**Authors:** E. B. Richardson, R. S. Hardenstine, K. A. O'Toole, A. J. McIvor, L. Calabrese, S. R. Laughlin, B. J. Scannell, J. E. M. Cochran, M. L. Berumen

**Affiliations:** ^1^ Division of Biological and Environmental Sciences and Engineering King Abdullah University of Science and Technology (KAUST) Thuwal Saudi Arabia; ^2^ Department of Environmental Protection and Regeneration Red Sea Global (RSG) Umluj Saudi Arabia; ^3^ Nujuma Ritz Carlton Reserve, Red Sea Global Ummahat Saudi Arabia; ^4^ School of Marine and Atmospheric Sciences Stony Brook University Southampton New York USA

**Keywords:** elasmobranchs, guitarfish, lagoon, osprey, predator–prey, Red Sea nursery, trophic interaction

## Abstract

The Halavi guitarfish (*Glaucostegus halavi*) is a Critically Endangered but poorly studied batoid found in the northwestern Indian Ocean. Its trophic ecology, both as predator and prey, remains largely undescribed. This note reports evidence of osprey ( *Pandion haliaetus haliaetus* ) predation on early lifestage Halavi guitarfish in the northern Red Sea. The six predation records presented here suggest that sheltering in shallow water may increase exposure to avian predation even as it reduces exposure to predation by other fish. Of these records, one represents direct photographic evidence of a successful predation attempt, showcasing an osprey carrying a guitarfish in its talons, while the remaining observations provide indirect evidence consistent with predation. The remains of two individuals were discovered in osprey nests, and two more individuals were found desiccated above the shoreline with organs removed. Another Halavi guitarfish, captured alive, exhibited wounds consistent with talon marks from an avian predation attempt. These occurrences were documented across six islands in the Al Wajh lagoon from 2020 through 2024 and suggest that osprey–elasmobranch interactions remain largely undocumented in the scientific literature, with most evidence currently emerging from opportunistic citizen‐science reports. Further investigation (potentially using biochemical markers such as stable isotopes) is needed to better understand the ecological implications of these events.

## Graphical abstract

We report six cases of suspected and confirmed osprey predation events on early lifestage Halavi guitarfish in the Al Wajh lagoon, northern Red Sea. These observations provide rare evidence of avian‐elasmobranch interactions in the region, and suggest that the use of shallow habitats may increase exposure to osprey predation. Together, these highlight a poorly documented risk for one of the most threatened batoid species.

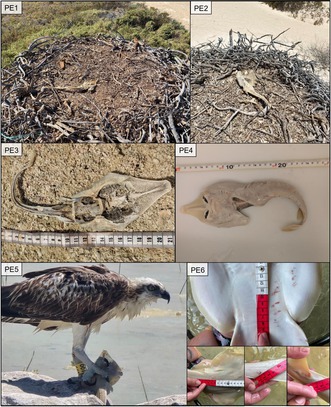

The Halavi guitarfish (*Glaucostegus halavi*, Forsskål 1775) is a regionally endemic batoid found in the northwestern Indian Ocean (Last et al. 2016). This species is listed as Critically Endangered on the IUCN Red List (Kyne and Jabado 2019), likely due to the targeted fishing of Rhinopristiformes across much of its range (Jabado 2018; Spaet and Berumen 2015). Despite growing conservation concern, most available data are limited to fish market records or opportunistic sightings (Garzon et al. 2022; Jabado 2018, 20; Moore 2012; Randall and Compagno 1995). Information on their trophic interactions, particularly as prey species for other predators, is lacking.

The Al Wajh lagoon is a shallow, semi‐enclosed bay spanning ~2080 km^2^ between the Saudi Arabian mainland on the east and an archipelago of 92 islands to the west (Figure 1; Chalastani et al. 2020). A recent study has identified this site as a critical habitat for multiple elasmobranch species (McIvor et al. 2026), particularly young‐of‐the‐year (YOY) Halavi guitarfish (IUCN SSC 2023). Regular field work targeting both avian and batoid species, has been conducted at this site since 2020. Guitarfish surveys were conducted by a team walking the shoreline, and corralling observed individuals into a seine net, where it was captured with dip nets, and transferred to a holding pool. Each individual was measured (total length, disc width, spiracle width, clasper lengths), sexed, and maturity classified where possible (Scannell unpubl. data). Animals were then tagged with a passive‐integrated‐transponder (PIT; BioMark) for identification purposes and released after approximately five minutes of handling. Similar expeditions targeting avifauna identified osprey breeding on 47 and 56 islands in 2021 and 2023 respectively (Calabrese et al. 2024). Tracking data from tagging efforts suggests that these individuals catch their prey mainly in shallow waters (Calabrese unpubl. data), and commonly in areas where early lifestage Halavi guitarfish have been observed. Guitarfish collections were conducted with approval from the Red Sea Zone Environmental Operations Department of Red Sea Global (RSZA‐EO‐FRM‐004), and ethically according to the Institutional Animal Care and Use Committee at King Abdullah University of Science and Technology protocol (#23IACUC010).

**FIGURE 1:**
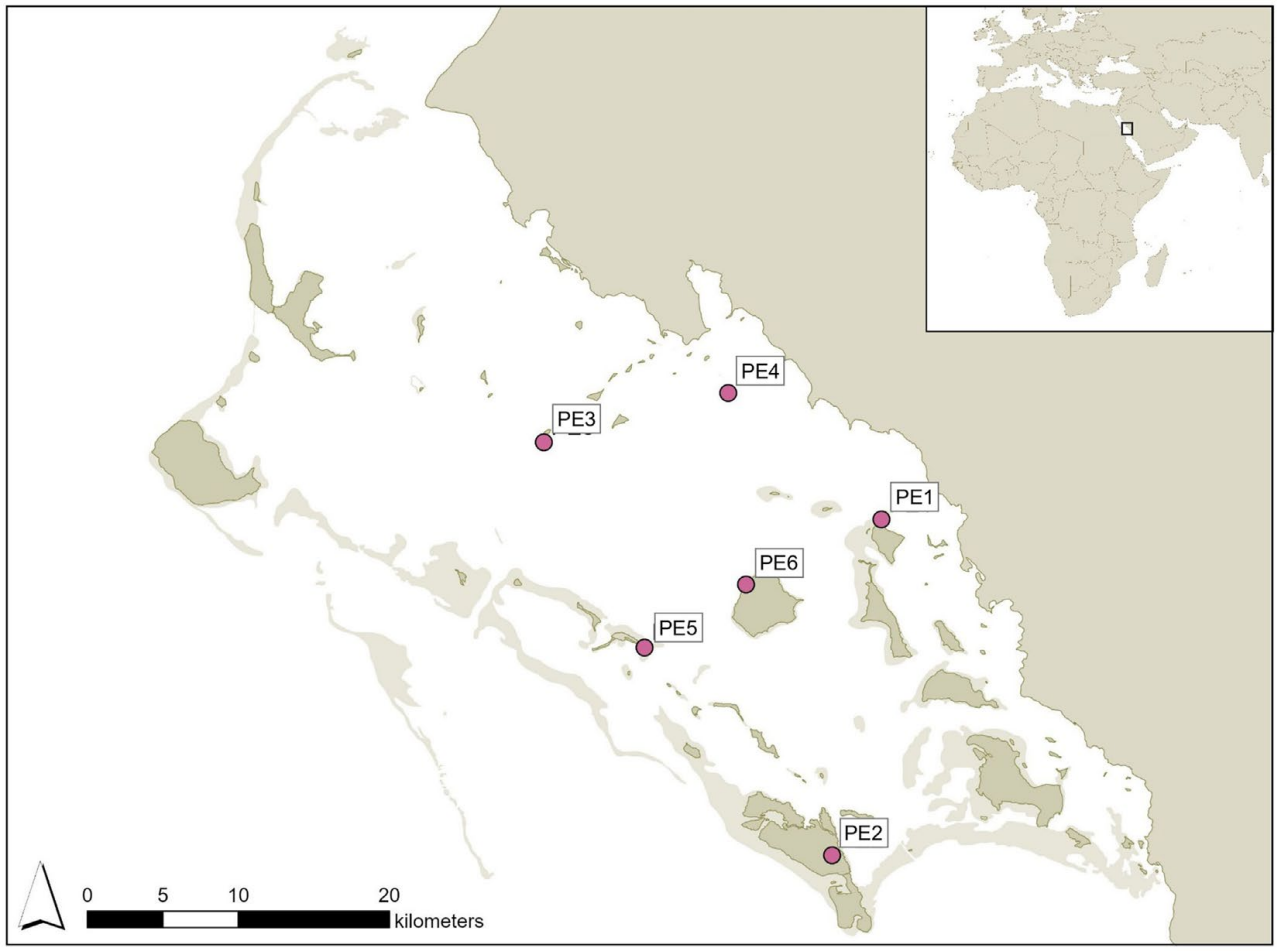
Map showing the Al Wajh lagoon and locations of potential predation events (PE) on Halavi guitarfish (*Glaucostegus halavi*) by osprey ( *Pandion haliaetus haliaetus* ) in chronological order.

During these surveys, opportunistic observations provided evidence of potential osprey‐guitarfish interactions, which were categorised as ‘inferred’ or ‘direct’ predation events (PEs) (Table 1; Figures 1 and 2). The latter was assigned to records in which an osprey was observed actively attempting to prey upon a guitarfish, while inferred PEs described circumstantial evidence and not a direct interaction.

**TABLE 1:** Details of observed evidence indicating predation events on juvenile Halavi guitarfish (*Glaucostegus halavi*) by osprey ( *Pandion haliaetus haliaetus* ), with characteristics such as total length (TL) and sex recorded.

ID#	Date	Time	Tides	Island	TL (cm)	Lifestage	Sex	PE	Description
PE1	1‐Dec‐2020	NA	NA	Mudra	50–60	JUV	NA	Inferred	Deceased, dried, found within osprey nest
PE2	30‐Jun‐2022	NA	NA	Sheybarah	40	YOY	NA	Inferred	Deceased, dried, found within osprey nest, some organ removal
PE3	15‐Aug‐2022	NA	NA	Al Osh Al Gharbi	28	YOY	NA	Inferred	Deceased, dried, found above shoreline, organ removal
PE4	26‐Oct‐2022	NA	NA	Al Diyar	33	YOY	M	Inferred	Deceased, found above shoreline, organ removal
PE5	01‐ Aug‐2024	1210	Low 06:30 High 15:00	Quman	NA	NA	NA	Direct	Clutched in talons of osprey, osprey flew away with guitarfish
PE6	31‐Oct‐2024	1138	Low 10:00 High 18:39	Ummahat	64.7	JUV	M	Inferred	Alive, superficial punctures on both ventral and dorsal side, including first dorsal fin, partial healing of scars

**FIGURE 2:**
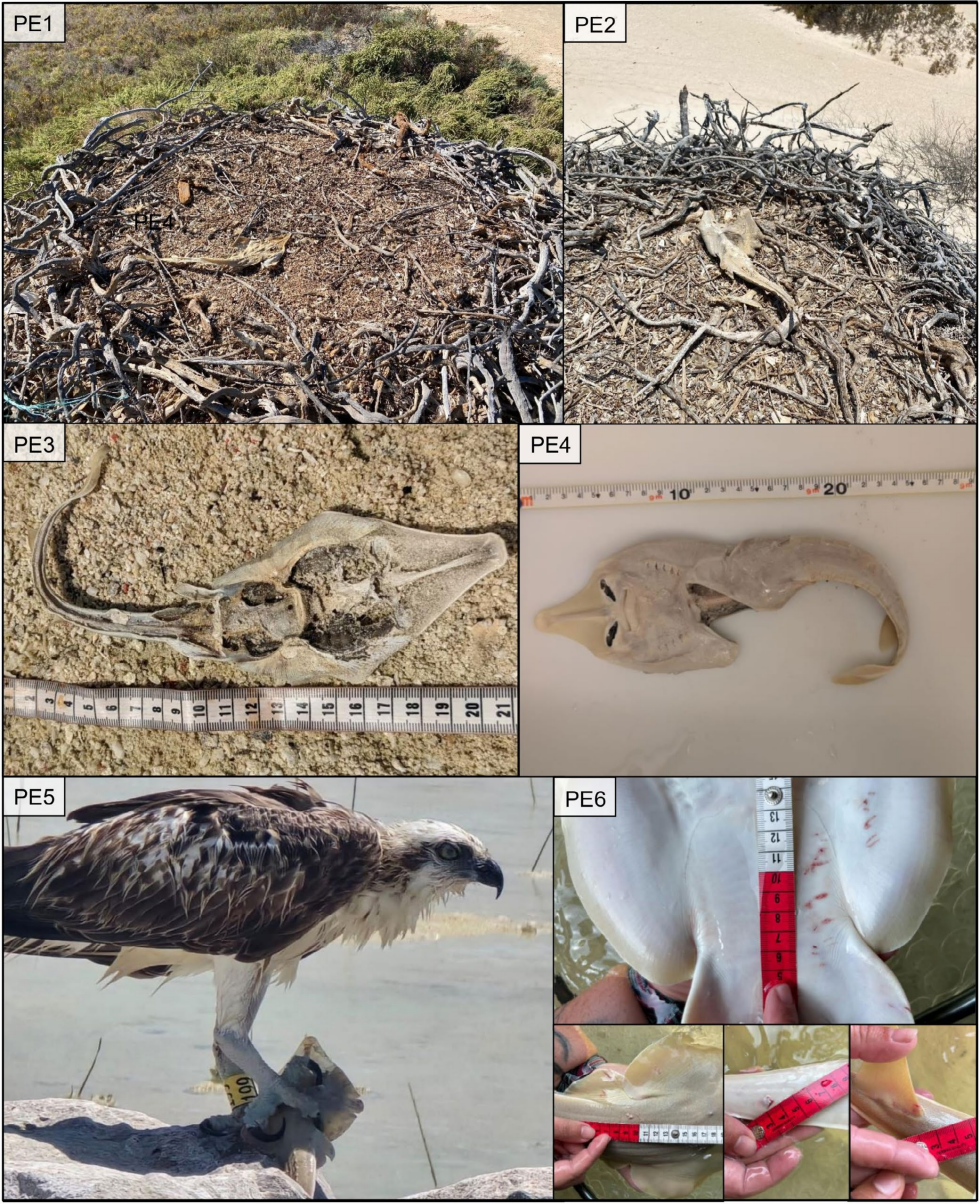
Photographic evidence of potential osprey predation events (PE) on Halavi guitarfish (*Glaucostegus halavi*) in the Al Wajh lagoon showing: (1–2) individuals found in osprey ( *Pandion haliaetus haliaetus* ) nests; (3–4) individuals observed above the tide line with internal organs missing; (5) a direct predation event; (6) an individual caught while seining that showcased superficial wounds possibly caused by osprey talons.

First, two early lifestage individuals were observed within osprey nesting sites during surveys (Figure 2; PE1–2), in December 2020 and June 2022, respectively. These individuals were both dried, with one (PE2) appearing to be half‐eaten. Two YOY animals were later found above the tideline with evidence of organ removal in August (Figure 2; PE 3) and October 2022 (Figure 2; PE4). The observation of a Palearctic osprey ( *Pandion haliaetus haliaetus* , Linnaeus 1758) clutching a YOY Halavi guitarfish in its talons (Figure 2, PE 5) provided more conclusive evidence of predation in August 2024. Finally, a captured Halavi guitarfish in October 2024 exhibited wounds consistent with superficial talon punctures (Figure 2, PE 6). These wounds did not appear to penetrate vital organs, and the guitarfish exhibited normal swimming behaviour with no apparent stress response to handling. This was the only instance of such scarring from 210 animals caught via seine net surveys, representing less than 0.05% of the sampled population.

Historical reports suggest that ospreys target fish that are 28 cm in length on average (Swenson 1978) and that bony fish make up the majority of their diet (Allen et al. 2018). This size preference corresponds to the total length (TL) of early lifestage guitarfish in the Al Wajh lagoon. The birth size of the Halavi guitarfish is around 28 cm (Last et al. 2016), though free swimming animals as small as 25 cm have been observed and YOY may range up to 40 cm (IUCN SSC 2023; McIvor et al. 2026), making them ideal food sources for local ospreys. This may also explain the survival of the guitarfish from PE6 at 64.7 cm TL (Table 1); it may have simply been too large for a successful predation attempt.

On islands, ospreys typically build large ground nests that are reused annually. Each year, additional materials, such as branches and other vegetation, are added to increase the nest's size and stability. Besides structural material, ospreys collect ornamental items such as dead corals, sponges, beach debris, and even dead birds as decorative elements in nests (Calabrese et al., unpublished data). The discovery of one individual (Figure 2; PE1) coincided with the beginning of the osprey breeding season (Calabrese et al. 2024). Although the individual appears almost intact, it is unclear whether it was collected for ornamental purposes or as a prey item consumed before breeding began. In contrast, another specimen (Figure 2; PE2) was half‐eaten and observed after the end of the breeding season (mid‐May; Calabrese et al. 2024), suggesting it may be a partially consumed prey item. In the case of all foraged guitarfish (PE1–4), a substantial portion of the body remained uneaten, which indicates that the edible fraction of this species, at least for osprey, is only a small proportion of its total mass.

Reports of Halavi guitarfish accidentally stranding during low tide elsewhere in the region (Whelan et al. 2018) add further uncertainty to these events. For most of these observations, it remains unclear whether the individuals were actively predated, scavenged after death, or encountered by ospreys following accidental stranding. Opportunistic hunting by inexperienced juveniles may also contribute to these observations. The osprey photographed carrying a guitarfish (Figure 2; PE5) was identified as a YOY (Calabrese et al. unpublished data), and raptors of this age are known to target easily available but often suboptimal prey as they are learning to hunt.

Ospreys typically eat the entire fish, with the exception of tougher parts such as the opercular bone, starting with the head (Clancy 2005). The harder outer layer of the guitarfish may encourage alternative feeding strategies, similar to other raptors that eviscerate prey from the softer underside (Keeley and Bechard 2017). Evisceration has been recorded in observations of gull predation on other batoids (Pleva 2009; Fitzsimons 2021) and appears to be a common strategy for hunting elasmobranchs in general (Engelbrecht et al. 2019; Higuera‐Rivas et al. 2025; Reeves et al. 2025). Both sharks and rays use the liver for lipid storage, making it by far the most nutrient dense portion of the body (Ballantyne 1997) and potentially explaining evisceration as a foraging strategy in predators targeting elasmobranchs.

Predation risk for early lifestage elasmobranchs is often elevated in shallow nursery habitats, where juveniles utilise shorelines to avoid larger in‐water predators (Doan and Kajiura 2020; Knip et al. 2010; Martins et al. 2020). Batoids, especially, can exploit extremely shallow water due to their flattened morphology, yet inexperienced individuals may be vulnerable to predation (Elston et al. 2017) and accidental stranding, therefore increasing the likelihood of avian predation. Spatial and tidal dynamics may also shape osprey‐guitarfish interactions; juvenile elasmobranchs commonly use these habitats around high tide (pers. obs.), while reports of tidal preference for osprey foraging are inconsistent (Flemming et al. 1992; Leurs et al. 2023). The direct predation observation (Figure 2; PE3) occurred shortly after low tide, while PE4 (Figure 2) was captured on a rising tide, but the timing of injury remains uncertain due to partial healing.

Collectively, these observations provide direct evidence of osprey predation in at least one instance, and multiple lines of indirect evidence suggesting additional interactions, highlighting a trophic dynamic between avian and elasmobranch species in the Red Sea. This being said, the Halavi guitarfish may constitute a suboptimal prey item for ospreys, hunted opportunistically when encountered. Avian predation on bony fishes has been well documented; however, elasmobranch records are limited (Ajemian et al. 2011; Bostic and Banks 1966; Fitzsimons 2021; Martin 2004). Osprey interactions with sharks and rays are lacking in the scientific literature, with only a single report published (Fernández‐Ordóñez et al. 2016). However, contextual observations reported on social media (Neith 2025; Allia 2025; Smith 2024; Sadrpour 2022) suggest that osprey–elasmobranch interactions may be globally underreported in peer‐reviewed articles.

Further investigation into the trophic ecology of the Al Wajh lagoon could provide valuable insight into osprey‐guitarfish interactions. Stable isotope analysis of local ospreys could provide quantitative evidence of the trophic linkage between ospreys and Halavi guitarfish within the lagoon and a better understanding of the tradeoffs between aquatic and avian predation risk in elasmobranchs.

In summary, this study provides the first documented evidence of osprey predation events on early lifestage Halavi guitarfish, with six independent potential interactions recorded in the Al Wajh lagoon, northern Red Sea. These observations highlight an underreported trophic dynamic and emphasise the importance of considering both in‐water and avian threats to vulnerable elasmobranch lifestages, especially in nursery habitats.

## Author Contributions

**E. B. Richardson:** conceptualization (equal), data curation (equal), investigation (lead), project administration (lead), visualization (equal), writing – original draft (lead). **R. S. Hardenstine:** conceptualization (equal), data curation (equal), investigation (equal), writing – review and editing (equal). **K. A. O'Toole:** conceptualization (equal), data curation (equal), investigation (equal), writing – review and editing (equal). **A. J. McIvor:** data curation (equal), project administration (equal), writing – review and editing (equal). **L. Calabrese:** data curation (equal), investigation (equal), writing – review and editing (equal). **S. R. Laughlin:** data curation (equal), writing – review and editing (equal). **B. J. Scannell:** investigation (equal), writing – review and editing (equal). **J. E. M. Cochran:** investigation (equal), supervision (equal), writing – review and editing (equal). **M. L. Berumen:** resources (equal), supervision (equal), writing – review and editing (equal).

## Funding

All field equipment was covered by the Reef Ecology Lab in association with King Abdullah University of Science and Technology (KAUST). Boat use and accommodation costs were covered by the Department of Environmental Protection and Regeneration, Red Sea Global.

## Conflicts of Interest

The authors declare no conflicts of interest.

## Data Availability

All data supporting the findings of this study are included within the manuscript, specifically in Table 1 and Figure 2.
